# Supplementary material for: Uncovering Genomic Features and Biosynthetic Gene Clusters in Endophytic Bacteria from Roots of the Medicinal Plant Alkanna tinctoria Tausch as a Strategy To Identify Novel Biocontrol Bacteria
Source: Microbiol Spectr. 2023 Jul 12;11(4):e00747-23. doi: 10.1128/spectrum.00747-23 (PMC10434035; doi:10.1128/spectrum.00747-23)
Supplement: Supplemental file 2 — Supplemental material. Download spectrum.00747-23-s0002.docx, DOCX file, 3.2 MB [file spectrum.00747-23-s0002.docx]

**Uncovering genomic features and biosynthetic gene clusters in endophytic bacteria from roots of the medicinal plant *Alkanna tinctoria* Tausch as a strategy to identify novel biocontrol bacteria**

Henry D. Naranjo ^a,1^**^*^**, Angélique Rat ^a,2^**^*^**, Noémie De Zutter ^b^, Emmelie De Ridder ^a^, Liesbeth Lebbe ^a^, Kris Audenaert ^b^, Anne Willems ^a,#^ .

^a^ Laboratory of Microbiology, Department of Biochemistry and Microbiology, Faculty of Sciences, Ghent University, Ghent, Belgium.

^b^ Laboratory of Applied Mycology and Phenomics, Department of Plants and Crops, Faculty of Bioscience Engineering, Ghent University, Ghent, Belgium.

^*^ These authors have contributed equally to this work and share the first authorship. Henry D. Naranjo is listed first, as he was responsible for coordinating the analysis and writing.

^1^ Present address: Facultad de Ciencias Químicas, Universidad Central del Ecuador, Quito 170521, Ecuador.

^2^ Present address: Bergelson Lab, Department of Biology, New York University, New York, USA.

**^#^ Correspondance:**

Professor Anne Willems

Anne.Willems@UGent.be

Running Title: Using genomic features to identify biocontrol bacteria.

**SUPPLEMENTARY FIGURES.**


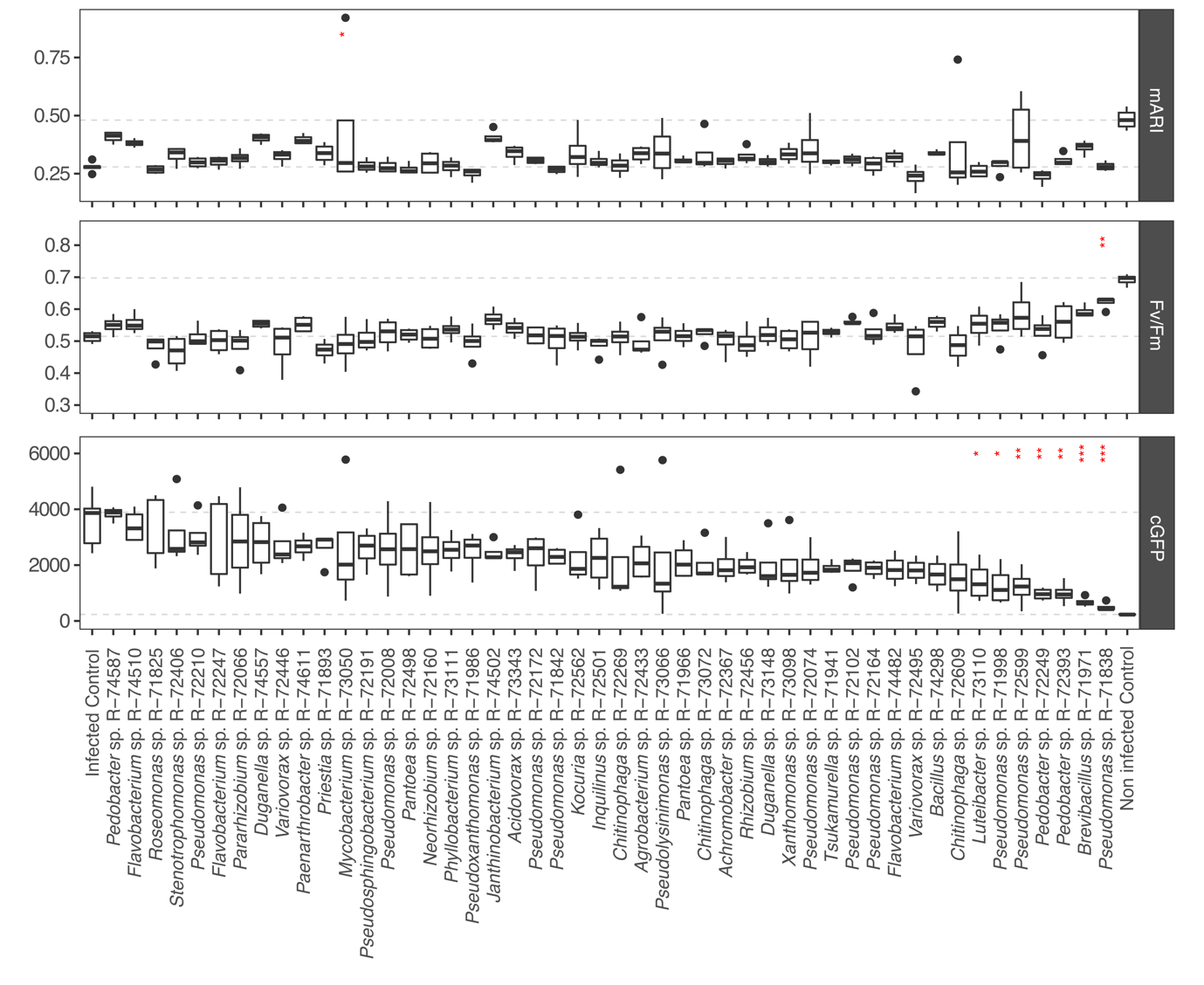


**Figure S1.** Boxplots showing the effect of all individual strains of the bacterial collection in the detached leaf assay. Responses shown are fungal biomass accumulation (cGFP), chlorophyll fluorescence (Fv/Fm), and estimation of anthocyanin content (mARI) on detached wheat leaves co-inoculated and incubated for a period of 72h with Fusarium graminearum PH-1 (n = 4 biological replicates). The non-infected control was inoculated with PBS. Significant differences from the infected control are indicated for p < 0.001 (***), p < 0.05 (**) and p < 0.01 (*); statistical differences were computed based on the Dunnett test.

**Figure S2.** Co-culture compatibility for the selected best-performing strains with antifungal effect. A: Brevibacillus sp. R-71971 vs. Pedobacter sp. R-72393, B: Brevibacillus sp. R-71971 vs. Pseudomonas sp. R-71838 and C: Pseudomonas sp. R-71838 vs. Pedobacter sp. R-72393.

**
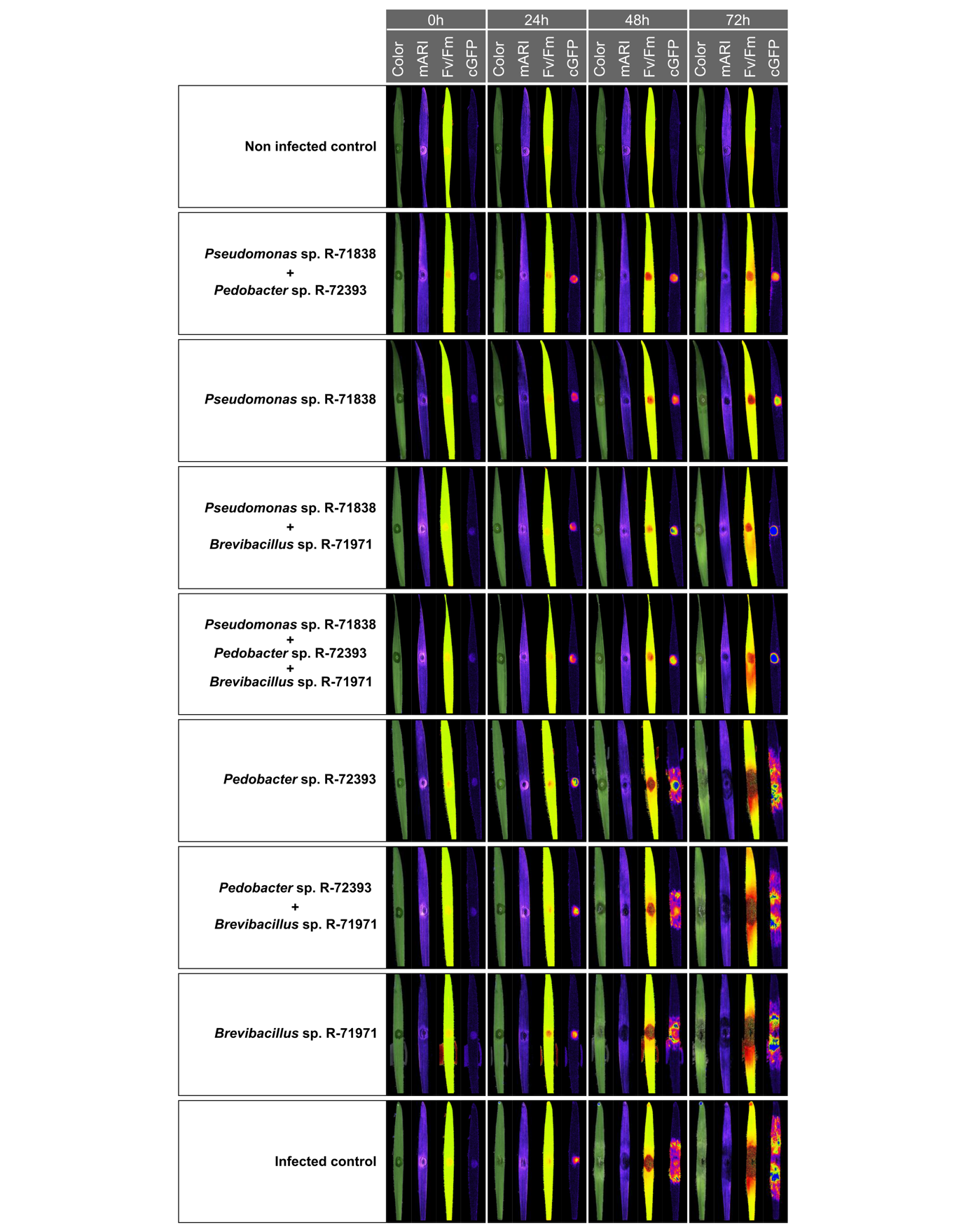
**

**Figure S3**. Overview of a subset of the multispectral images (Color, Chlorophyll fluorescence, cGFP and mARI) at different time points (0h, 24h, 48h, 72h) for the best-performing strains and combinations in the detached leaf assay. The infected and non-infected controls were included as a reference. The non-infected control was inoculated with PBS only.


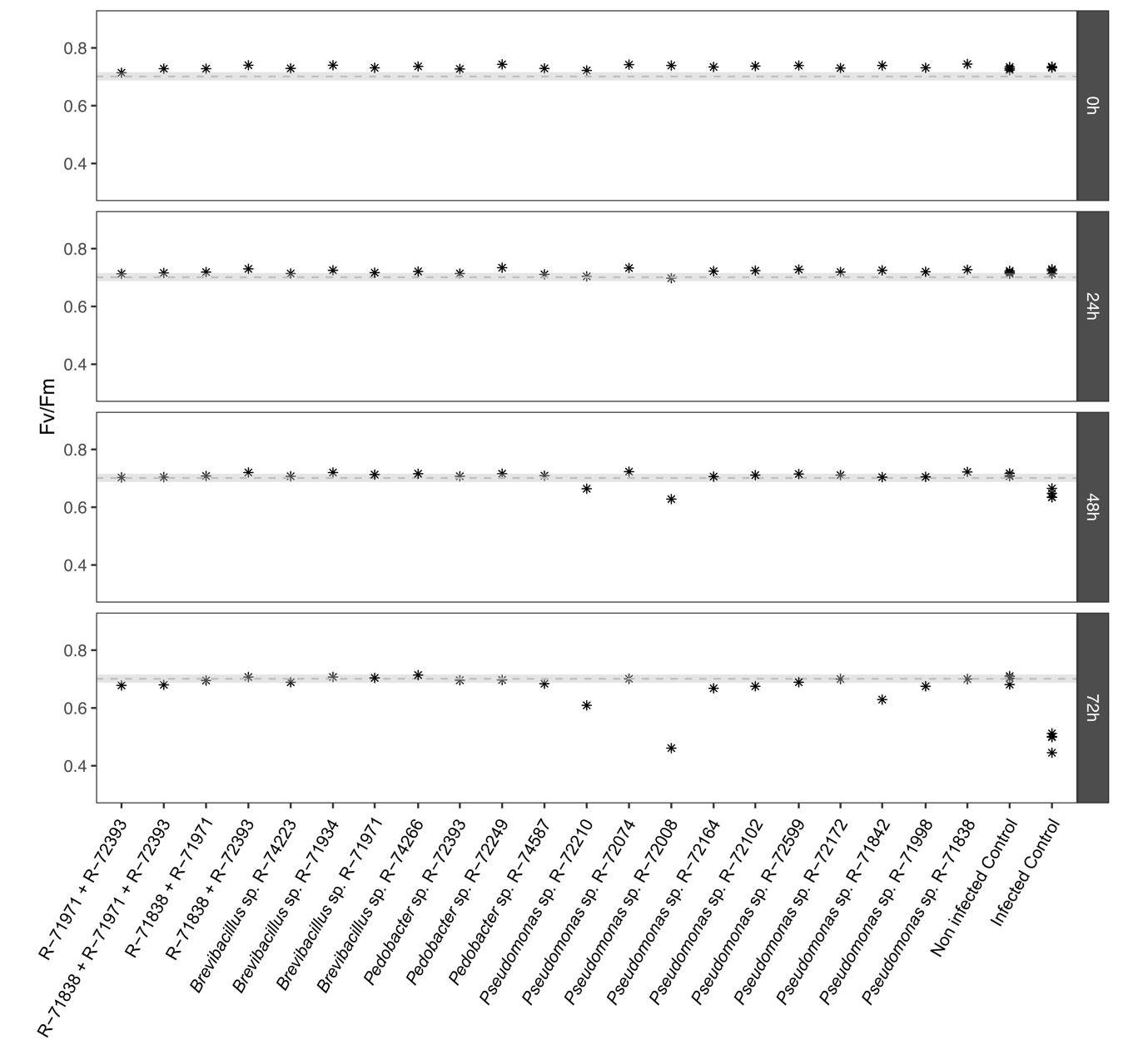


**Figure S4.** Evaluation of the potential phytotoxic effect of selected bacterial treatments and combinations (SynCom experiment) based on chlorophyll fluorescence (Fv/Fm) on detached wheat leaves, incubated at different time points (0, 24, 48, 72h). Treatments were inoculated with the bacteria suspended in PBS (n = 1 biological replicate), non-infected controls were inoculated with PBS alone (n = 3 biological replicates), and infected controls were inoculated with Fusarium graminearum PH-1 suspended in PBS (n = 3 biological replicates).


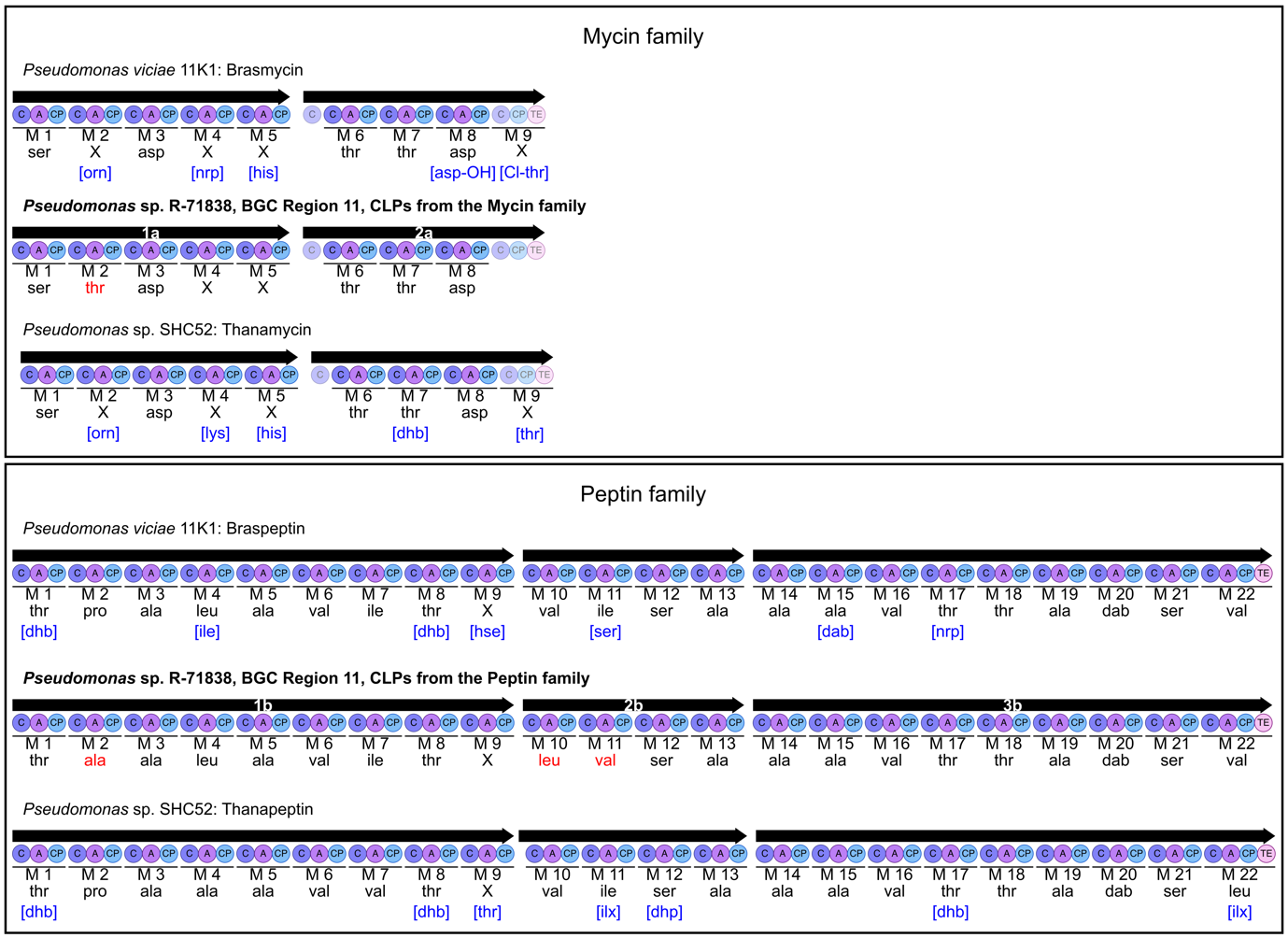


**Figure S5.** Nonribosomal peptides (NRPs) sequences for cyclic lipopeptides (CLPs) as predicted in region 11 from Pseudomonas sp. R-71838. Genes 1a and 2a are likely associated with the biosynthesis of a CLP from the mycin family. Whereas genes 1b, 2b and 3b are likely associated with the peptin family. A minimal module of the nonribosomal peptides consists of three domains: a condensation (C) domain, an adenylation (A) domain, and a carrier protein [CP] domain. The aminoacidic sequence of the different peptides is predicted by antiSMASH; X denotes residues without prediction. Residues highlighted in blue are denoted to differ from the in silico prediction and represent the original descriptions. Amino acids highlighted in red represent unique residues that define novel variants of peptin and mycin CLPs from Pseudomonas R-71838.

**SUPPLEMENTARY TABLES.**

**Table S1.** Taxonomic identification and genomic features of 88 bacterial strains were included in this study. Genomic data were deposited in the IMG database, and accession codes were indicated as IMG IDs. Genome-based identification was made with GTDB-Tk v2.0.0, and CheckM v1.1.3 was used to evaluate the quality of assemblies and estimate the size; all genomes had a minimum completeness of 95% and contamination of less than 5%. BGCs were calculated with antiSMASH. *Illumina sequencing was used for most of the assemblies, except for these selected genomes assembled with hybrid technology (Nanopore + Illumina); contig numbers in parenthesis indicate those pre-assemblies using Illumina data only, as well as its corresponding BGCs prediction.

| **Class** | **Strain code** | **Identification** | **IMG ID** | **Size (Mbp)** | **Contigs** | **BGCs** |
| --- | --- | --- | --- | --- | --- | --- |
| Alpha-proteobacteria | R-72160 | *Neorhizobium cellulosilyticum* | 2857531043 | 6.75 | 81 | 10 |
|  | R-72433* | *Agrobacterium* *divergens* | 2929138655 | 5.81 | 3 (23) | 4 (4) |
|  | R-72456 | *Rhizobium* sp. | 2857516855 | 7.79 | 89 | 14 |
|  | R-72066 | *Pararhizobium* sp. | 2842922631 | 5.82 | 44 | 9 |
|  | R-73111 | *Phyllobacterium* sp. | 2842871566 | 4.83 | 30 | 3 |
|  | R-73088 | *Bradyrhizobium* sp. | 2857509624 | 7.47 | 36 | 7 |
|  | R-73074 | *Tardiphaga robiniae* | 2857524615 | 6.62 | 25 | 5 |
|  | R-72139 | *Methylobacterium* sp. | 2842698319 | 5.19 | 84 | 10 |
|  | R-72369 | *Methylopila* sp. | 2842694124 | 4.06 | 313 | 4 |
|  | R-72291 | *Caulobacter* sp. | 2857504554 | 5.37 | 23 | 4 |
|  | R-71825 | *Roseomonas aerophila* | 2842775625 | 5.59 | 51 | 8 |
|  | R-73070* | *Roseomonas hellenica* | 2929199973 | 7.26 | 2 (237) | 9 (12) |
|  | R-72501 | *Inquilinus* sp. | 2844533157 | 7.52 | 276 | 9 |
| Beta-proteobacteria | R-74599 | *Duganella* sp. | 2857564685 | 6.29 | 20 | 6 |
|  | R-74557 | *Duganella* sp. | 2857553236 | 6.17 | 44 | 4 |
|  | R-74565 | *Duganella* sp. | 2857558681 | 6.62 | 96 | 8 |
|  | R-73148 | *Duganella* sp. | 2842711865 | 7.16 | 49 | 6 |
|  | R-74502 | *Janthinobacterium lividum* | 2857547612 | 6.18 | 35 | 8 |
|  | R-72367 | *Achromobacter aestuarii* | 2857542790 | 5.33 | 9 | 8 |
|  | R-71975 | *Achromobacter* sp. | 2857537821 | 5.25 | 48 | 3 |
|  | R-72090 | *Pigmentiphaga* sp. | 2857576091 | 5.47 | 51 | 6 |
|  | R-72349* | *Variovorax* sp. | 2929160207 | 9.08 | 3 (93) | 10 (13) |
|  | R-72016* | *Variovorax* sp. | 2929168669 | 9.13 | 2 (43) | 10 (10) |
|  | R-72495 | *Variovorax* sp. | 2842677519 | 5.62 | 110 | 11 |
|  | R-72446 | *Variovorax* sp. | 2842733646 | 5.72 | 37 | 6 |
|  | R-72060 | *Variovorax* sp. | 2842747753 | 5.58 | 39 | 6 |
|  | R-73343 | *Acidovorax* sp. | 2842718218 | 4.56 | 20 | 4 |
|  | R-71998 | *Pseudomonas fluorescens* | 2842854478 | 6.14 | 47 | 11 |
|  | R-71838* | *Pseudomonas* sp. | 2929144301 | 6.62 | 1 (61) | 16 (22) |
|  | R-72074 | *Pseudomonas fluorescens* | 2842843487 | 6 | 23 | 10 |
|  | R-72164 | *Pseudomonas atacamensis* | 2842832357 | 5.96 | 20 | 9 |
| Gamma-proteobacteria | R-72102 | *Pseudomonas paracarnis* | 2842837860 | 6.07 | 59 | 18 |
|  | R-72172 | *Pseudomonas paracarnis* | 2842826826 | 5.97 | 59 | 17 |
|  | R-71842* | *Pseudomonas orientalis* | 2929189879 | 5.93 | 1 (25) | 16 (16) |
|  | R-72008 | *Pseudomonas viridiflava* | 2842849001 | 5.92 | 37 | 8 |
|  | R-72210 | *Pseudomonas viridiflava* | 2842815866 | 5.95 | 46 | 8 |
|  | R-72599 | *Pseudomonas* sp. | 2842805378 | 5.39 | 11 | 7 |
|  | R-72498 | *Pantoea agglomerans* | 2844528606 | 4.73 | 26 | 7 |
|  | R-71966 | *Pantoea agglomerans* | 2865014394 | 4.76 | 47 | 8 |
|  | R-74235 | *Stenotrophomonas maltophilia* | 2857442823 | 4.56 | 55 | 4 |
|  | R-72406 | *Stenotrophomonas* sp. | 2842757796 | 3.98 | 16 | 2 |
|  | R-73098* | *Xanthomonas* sp. | 2929195423 | 5.33 | 1 (50) | 5 (11) |
|  | R-71986 | *Pseudoxanthomonas* sp. | 2842780639 | 4.34 | 6 | 3 |
|  | R-72151 | *Luteibacter* sp. | 2842914999 | 4.42 | 29 | 11 |
|  | R-73110 | *Luteibacter* sp. | 2842918807 | 4.29 | 6 | 7 |
| Sphingobacteria | R-72249 | *Pedobacter* sp. | 2842722452 | 6.26 | 21 | 6 |
|  | R-72393 | *Pedobacter* sp. | 2842909656 | 6.19 | 35 | 6 |
|  | R-74587 | *Pedobacter* sp. | 2857627736 | 5.63 | 42 | 7 |
|  | R-72191 | *Pseudosphingobacterium* sp. | 2842903701 | 6.99 | 33 | 12 |
| Flavobacteria | R-74510* | *Flavobacterium* sp. | 2929150217 | 5.46 | 1 (39) | 7 (7) |
|  | R-74482 | *Flavobacterium* sp. | 2857618242 | 5.64 | 26 | 7 |
|  | R-72247 | *Flavobacterium* sp. | 2857613821 | 4.92 | 23 | 9 |
| Chitinophagia | R-73072* | *Chitinophaga* sp. | 2929239360 | 7.75 | 1 (30) | 16 (19) |
|  | R-72609* | *Chitinophaga* sp. | 2929921140 | 8.65 | 1 (42) | 24 (28) |
|  | R-72269* | *Chitinophaga* sp. | 2929177148 | 7.88 | 1 (19) | 13 (10) |
|  | R-72421* | *Filimonas* sp. | 2929154850 | 6.75 | 1 (23) | 3 (3) |
| Bacilli | R-72492 | *Peribacillus frigoritolerans* | 2842682962 | 5.59 | 35 | 9 |
|  | R-71875 | *Peribacillus frigoritolerans* | 2849139964 | 5.61 | 46 | 8 |
|  | R-71922 | *Peribacillus* sp. | 2857581216 | 5.52 | 35 | 12 |
|  | R-71921 | *Domibacillus* sp. | 2857604169 | 5.11 | 78 | 5 |
|  | R-71929 | *Domibacillus* sp. | 2857609550 | 3.75 | 41 | 3 |
|  | R-71935 | *Gottfriedia* sp. | 2857586860 | 4.35 | 33 | 6 |
|  | R-74298* | *Bacillus cereus* | 2929233124 | 5.95 | 5 (59) | 11 (13) |
|  | R-71893 | *Priestia megaterium* | 2842882022 | 6.16 | 64 | 8 |
|  | R-74266 | *Brevibacillus* sp. | 2857465823 | 6.77 | 54 | 11 |
|  | R-71934 | *Brevibacillus* sp. | 2857591370 | 6.57 | 29 | 9 |
|  | R-71971* | *Brevibacillus* sp. | 2929183550 | 6.38 | 2 (45) | 14 (15) |
|  | R-74223 | *Brevibacillus borstelensis* | 2857460504 | 5.19 | 146 | 9 |
|  | R-74146* | *Paenibacillus* sp. | 2929206907 | 5.92 | 1 (178) | 9 (11) |
|  | R-74130 | *Paenibacillus* sp. | 2857453340 | 8.09 | 55 | 7 |
|  | R-72005 | *Paenibacillus* sp. | 2864997549 | 5.14 | 68 | 8 |
|  | R-74131 | *Paenibacillus* sp. | 2865002811 | 6.33 | 90 | 11 |
|  | R-74144 | *Cohnella* sp. | 2857472729 | 6.57 | 103 | 7 |
| Actinomycetia | R-73066 | *Pseudolysinimonas* sp. | 2857737099 | 3.1 | 103 | 2 |
|  | R-73062 | *Rhodoglobus* sp. | 2857733635 | 3.53 | 12 | 3 |
|  | R-72288 | *Plantibacter* sp. | 2857729791 | 4.04 | 13 | 6 |
|  | R-72356 | *Microbacterium* sp. | 2857723135 | 4.22 | 26 | 3 |
|  | R-72113 | *Microbacterium* sp. | 2857720070 | 3.19 | 67 | 8 |
|  | R-73081 | *Micrococcus luteus* | 2857632687 | 2.45 | 288 | 6 |
|  | R-74225 | *Micrococcus luteus* | 2857479173 | 2.47 | 284 | 6 |
|  | R-72562 | *Kocuria rhizophila* | 2857727296 | 2.75 | 70 | 4 |
|  | R-74611 | *Paenarthrobacter* sp. | 2857740372 | 4.78 | 45 | 9 |
|  | R-73093 | *Brevibacterium* sp. | 2857710386 | 3.19 | 93 | 4 |
|  | R-74106 | *Nocardioides* sp. | 2857481737 | 4.76 | 204 | 3 |
|  | R-75348* | *Micromonospora* sp. | 2929219909 | 6.98 | 1 (338) | 11 (11) |
|  | R-74116* | *Micromonospora* sp. | 2929226422 | 7.25 | 1 (162) | 14 (15) |
|  | R-73050* | *Mycobacterium* sp. | 2929212328 | 7.71 | 3 (281) | 16 (19) |
|  | R-71941 | *Tsukamurella* sp. | 2842888712 | 4.28 | 39 | 14 |

**Table S2.** Pairwise comparison of the SynComs to the single strains included in the SynComs as evaluated in the detached leaf assay, as per ANOVA and post-hoc Dunnett-test at a 95%-significance level. Fv/Fm, chlorophyll fluorescence; mARI, modified anthocyanin reflectance index; cGFP, corrected GFP-signal; *, p < 0.05; **, p < 0.01; ***, p < 0.001; ns, not significant.

|  |  | Fv/Fm | | | |  | mARI | | | |  | cGFP | | | |
| --- | --- | --- | --- | --- | --- | --- | --- | --- | --- | --- | --- | --- | --- | --- | --- |
|  | Mean  ± sd. | R-71971  + R-71838 | R-71971  + R-72393 | R-72393  + R-71838 | R-71971  + R-71838  + R-72393 | Mean  ± sd. | R-71971  + R-71838 | R-71971  + R-72393 | R-72393  + R-71838 | R-71971  + R-71838  + R-72393 | Mean  ± sd. | R-71971  + R-71838 | R-71971  + R-72393 | R-72393  + R-71838 | R-71971  + R-71838  + R-72393 |
| R-71971 | 0.513  ± 0.026 | *** | ns |  | *** | 0.337  ± 0.019 | *** | ns |  | *** | 3158  ± 657 | ** | ns |  | * |
| R-71838 | 0.668  ± 0.005 | ns |  | ns | ns | 0.484  ± 0.014 | ns |  | ns | ns | 919  ± 169 | ns |  | ns | ns |
| R-72393 | 0.532  ± 0.009 |  | ns | *** | ** | 0.411  ± 0.018 |  | ns | ns | ** | 2463  ± 557 |  | ns | ** | ns |
| R-71971  + R-71838 | 0.653  ± 0.003 |  | *** | ns | ns | 0.469  ± 0.029 |  | * | ns | ns | 1185  ± 374 |  | * | ns | ns |
| R-71971  + R-72393 | 0.515  ± 0.022 | *** |  | *** | *** | 0.382  ± 0.010 | * |  | ** | *** | 2846  ± 342 | * |  | ** | * |
| R-72393  + R-71838 | 0.672  ± 0.007 | ns | *** |  | ns | 0.479  ± 0.017 | ns | ** |  | ns | 708  ± 88 | ns | ** |  | ns |
| R-71971  + R-71838  + R-72393 | 0.627  ± 0.018 | ns | ** | ns |  | 0.524  ± 0.013 | ns | *** | ns |  | 1223  ± 249 | ns | ns | ns |  |

**Table S3.** Details of BGCs predicted by antiSMASH for the best-performing strain (Pseudomonas sp. R-71838) and the 9 other Pseudomonas strains studied here. *Indicates BGCs unique for Pseudomonas sp. R-71838 that might contribute to the beneficial effect of this bacteria, a comparison of BGCs was made with BiG-SCAPE. The 16 regions described by antiSMASH correspond to the gene loci predicted to encode for BGCs. Furthermore, the prediction of the type of secondary metabolite and its similarity with other described BGCs are represented. The similarity with known BGCs is calculated based on amino acid composition.

| **Region** | **Type** | **Most similar known cluster** | **Similarity** | |
| --- | --- | --- | --- | --- |
| Region 1 | NRPS-like | fragin | NRP | 37% |
| Region 2 | arylpolyene | APE Vf | Other | 40% |
| Region 3 | RiPP-like |  |  |  |
| Region 4 | NAGGN |  |  |  |
| Region 5 | NRPS | pyoverdin | NRP | 10% |
| Region 6* | redox-cofactor | lankacidin C | NRP + Polyketide | 13% |
| Region 7* | ranthipeptide | pyoverdin | NRP | 8% |
| Region 8* | NRPS-like,T1PKS,NRPS | crochelin A | NRP + Polyketide | 7% |
| Region 9* | RiPP-like |  |  |  |
| Region 10* | NRPS | coelibactin | NRP | 27% |
| Region 11* | Homoserine lactone,NRPS | syringomycin | NRP | 100% |
| Region 12 | butyrolactone |  |  |  |
| Region 13 | betalactone | fengycin | NRP | 13% |
| Region 14* | NRPS | pyoverdin | NRP | 16% |
| Region 15 | redox-cofactor | lankacidin C | NRP + Polyketide | 13% |
| Region 16* | lanthipeptide-class-ii |  |  |  |

**Table S4.** Closest matched BGCs predicted by cblaster and ClusterBlast (antiSMASH) for unique clusters from Pseudomonas sp. R-71838. Matched MIBIGs and known secondary metabolites are predicted as well.

| **BGCs from *Pseudomonas* sp. R-71838** | **Type** | **Strains with the closest BGCs match** | **MIBIGs match** | **Predicted Product** |
| --- | --- | --- | --- | --- |
| Region 6 | redox-cofactor | *Pseudomonas ogarae* ACN4,  *Pseudomonas grimontii* BIGb0428,  *Pseudomonas* sp. St290 | - | - |
| Region 7 | ranthipeptide | *Pseudomonas* sp. 22 E 5,  *Pseudomonas viridiflava* p11.H11,  *Pseudomonas* sp. CG7 | - | - |
| Region 8 | NRPS-like,T1PKS,NRPS | *Pseudomonas brassicacearum* NFM421,  *Pseudomonas fluorescens* FW300-N2C3,  *Pseudomonas thivervalensis* DSM 13194 | BGC0002422 | Histicorrugatin |
| Region 9 | RiPP-like | *Pseudomonas* sp. SWRI179,  *Pseudomonas* sp. CG7,  *Pseudomonas thivervalensis* PITR2 | - | - |
| Region 10 | NRPS | *Pseudomonas* sp. CG7,  *Pseudomonas zanjanensis* SWRI12 1,  *Pseudomonas thivervalensis* BS3779 | - | - |
| Region 11 | Homoserine lactone,NRPS | *Pseudomonas* sp. SHC52,  *Pseudomonas viciae* 11K1,  *Pseudomonas* sp. In5 | - | - |
| Region 14 | NRPS | *Pseudomonas* sp. CG7,  *Pseudomonas thivervalensis* DSM 13194,  *Pseudomonas bijieensis* SP1 | BGC0002693 | Pyoverdine Pf-5 |
| Region 16 | lanthipeptide-class-ii | *Pseudomonas zanjanensis* SWRI12 5,  *Pseudomonas* sp. NFACC13-1,  *Pseudomonas* sp. C3-2018 | - | - |
